# Mismatch between actual and preferred extent of telework: cross-sectional and prospective associations with well-being and burnout

**DOI:** 10.1186/s12889-023-16683-8

**Published:** 2023-09-06

**Authors:** Marina Heiden, David M. Hallman, Malin Svensson, Svend Erik Mathiassen, Sven Svensson, Gunnar Bergström

**Affiliations:** 1 Centre for Musculoskeletal Research, Department of Occupational Health Science and Psychology, Faculty of Health and Occupational Studies, University of Gävle, SE 801 76 Gävle, Gävle, Sweden; 2 Unit of Intervention and Implementation Research for Worker Health, Institute of Environmental Medicine, Karolinska Institutet, Box 210, 171 77 Stockholm, Sweden

**Keywords:** Telecommuting, Flexible work, Person-environment fit, Match, Sweden

## Abstract

**Background:**

This study aimed to determine whether telework mismatch, *i.e.*, lack of fit between actual and preferred extent of telework, is cross-sectionally and prospectively associated with well-being and burnout.

**Methods:**

A questionnaire was sent to employees in a Swedish manufacturing company in November 2020 (baseline) and September 2021 (follow-up). It contained questions about well-being (WHO-5 Well-Being Index) and burnout (Copenhagen Psychosocial Questionnaire III), as well as the preferred extent of telework and extent of telework performed. Telework mismatch was calculated as the difference between the actual and preferred extent of telework. Change in mismatch over time was categorized as 1) less mismatch at follow-up than at baseline, 2) more mismatch at follow-up, and 3) identical levels of mismatch at baseline and follow-up. Multivariate and univariate analyses of variance were used to determine the effects of mismatch and change in mismatch over time on baseline ratings and changes in ratings of well-being and burnout. All analyses were performed with and without adjustment for age, sex, marital status, children, type of employment, commuting time and extent of telework performed.

**Results:**

The response rate was 39% at baseline (*n* = 928, 67% men, mean(SD) age: 45(11) years) and 60% at follow-up (*n* = 556, 64% men, mean(SD) age: 46(11) years). A cross-sectional association was found between telework mismatch and well-being, showing that employees who teleworked more than they would like reported worse well-being than those who teleworked less than they would like. No statistically significant association was found between telework mismatch and burnout. The ability of telework mismatch at baseline to predict changes in well-being or burnout over 10 months was small and non-significant. No association was found between change in telework mismatch over the 10-month period and corresponding changes in well-being or burnout.

**Conclusion:**

Our results suggest that telework should be thoughtfully practiced in companies/organizations to avoid negative consequences for employees who already telework more than they prefer. Studies are needed to determine how long-term changes in match between preferred and actual extent of telework is associated with employee well-being, including how the association is modified by the nature of the job and the work environment.

## Background

Telework is not a new phenomenon, but it received a dramatic increase in attention during the COVID-19 pandemic. Although telework can have different names and, to some extent, different definitions, it is usually considered to be a work practice involving working away from the regular office location using technology as needed to conduct the work tasks [1, 2]. During the pandemic, telework became practically synonymous with working from home, because of the restrictions implemented to reduce the spread of the SARS-CoV-2 virus. OECD reported increased rates of telework across countries worldwide, but also a large variation between countries in the extent of the increase [3]. In the European Union, the proportion of people teleworking increased from about 15% in 2019 to 48% in 2020 [4, 5].

In Sweden, just over 40% of workers started teleworking because of COVID-19 [6]. The government did not enforce any lockdowns but issued recommendations about *e.g.*, staying at home if experiencing symptoms, working from home and avoiding public transport if possible, and keeping distance to others [7, 8]. Schools and kindergartens remained open, but distance teaching was introduced in high schools and universities [7].

Previous studies of telework and well-being generally show that teleworking is beneficial for employees [9–13]. Benefits include greater autonomy that may increase job satisfaction and decrease emotional exhaustion. However, teleworking may also have negative effects, such as isolation that can decrease job satisfaction and performance. Two recent reviews by Beckel and Fisher [14] and Lunde et al. [1, 2] summarize studies performed prior to as well as during the COVID-19 pandemic. Beckel and Fisher [14] concluded that a beneficial association between telework and well-being likely requires that job resources are sufficient, and that telework arrangements are designed to fit individual needs. They also emphasized that studies on outcomes related to telework during the pandemic may be confounded by other exposures associated with working from home. Lunde et al. [1, 2] included only studies on teleworking from home, and attempts were made to reduce bias by excluding studies on populations that were exposed to very strict COVID-19 regulations. Due to the small number of included studies and their methodological weaknesses, no firm conclusions could be drawn about associations between teleworking from home and employee health. Both reviews concluded that more research is needed in this area.

According to person-environment fit theory, the degree of compatibility between an individual and his/her work environment affects outcomes such as job satisfaction and well-being [15–17]. If the characteristics of the individual and the work environment are well matched, the outcomes are positive [17]. One aspect of the fit is job content, including the work tasks performed. The person-environment-occupation model [18, 19] emphasizes the importance of the dynamic interaction between the individual, the work environment and the work-related activities for a person’s experience over time. In a telework context, employees who telework exactly to the extent they want may experience their work situation differently than employees who cannot telework as much as they would like, or employees who are forced to telework more than they would like. In support of this, Otsuka et al. [20] found that workers who preferred to telework experienced less psychological distress than workers who preferred not to telework when teleworking > 4 days per week. De Wind et al. [21] concluded that mismatch between employees’ access to and need for working from home was cross-sectionally associated with higher work-home interference and fatigue, while the mismatch was not associated with changes in the outcomes after one year.

When investigating the relationship between health-related outcomes and telework mismatch, *i.e.*, the difference between actual and preferred extent of telework, temporal aspects should be considered [18, 22]. Employees may change their preferences over time, especially during periods of intensified telework such as the COVID-19 pandemic, and these changes may be associated with employee well-being. For example, if the extent of telework performed is approaching the preferred extent of telework, employees may experience more well-being than if the extent of telework is moving in the opposite direction. Considering that changes in telework practices brought on by the COVID-19 pandemic [23] may to some extent persist in the future, it is important to understand the consequences they may have for employee well-being. Since COVID-19 restrictions in Sweden allowed for employees to work from home as well in the office, to the extent that they could still prevent spread of infection [24], data collected in Sweden may be particularly relevant in a study of telework mismatch.

The aim of the present study was to determine whether telework mismatch, *i.e.*, the difference between actual and preferred extent of telework, is cross-sectionally and prospectively associated with employee well-being and burnout among white collar employees. The specific research questions were:

1. To what extent do ratings of well-being and burnout differ between employees who telework as much as they would like (*i.e.*, have no mismatch), employees who telework less than they would like, and employees who telework more than they would like?
2. To what extent does telework mismatch predict changes in well-being and burnout over 10 months?
3. To what extent is change in telework mismatch over 10 months associated with changes in ratings of well-being and burnout?

## Methods

### Study design and participants

This study is part of the FLOC cohort study [25], and used a prospective design with two measurements 10 months apart. In November 2020, a questionnaire was distributed through e-mail to all 2373 white-collar employees in a Swedish company manufacturing energy supply systems, together with information about the study. At that time, national recommendations about working from home if possible had been in effect for approximately eight months [24]. In September 2021, when the recommendations were still in effect, a follow-up questionnaire was sent to the same employees. Qualtrics XM© software (Qualtrics, Provo, UT) was used to collect the questionnaire answers. All white-collar employees were eligible for the study since telework arrangements were common among the company's white-collar staff, and the company expressed interest in the research questions. The study was approved by the Swedish Ethical Review Authority (Ref. no. 2019–06220).

### Data collection and processing

At each measurement occasion, three reminders to answer the questionnaire were sent, approximately one week apart. The company provided e-mail addresses to the employees, together with information about their age, sex, type and extent of employment. The questionnaire was offered in Swedish or English, and contained validated questions about well-being (WHO-5 Well-Being Index, [26, 27]) and burnout (Copenhagen Psychosocial Questionnaire III, [28, 29]). In addition, questions were included about the respondent’s family situation, commuting time, preferred extent of telework and extent of telework performed. A description of each instrument and the independent variables *telework mismatch* and *change in telework mismatch* is provided below.

### Dependent variables

#### Well-being

Well-being was assessed by the WHO-5 Well-Being Index [26, 27], which contains five items about feelings over the past two weeks (*e.g.*, “I have felt cheerful and in good spirits”, “I have felt calm and relaxed”). Responses were given on 6-point scales ranging from “at no time” to “all of the time”. They were scored from 0 to 100 in steps of 20, so that higher values indicate better well-being. If at least three of the items had been responded to, a mean value was calculated from the scores. Thus, the total score ranged from 0 to 100 where higher values indicate better well-being. The internal consistency of the scale, in terms of Cronbach’s alpha in the sample, was 0.89 at baseline and 0.88 at follow-up.

#### Burnout

Burnout was assessed by the burnout scale in the Copenhagen Psychosocial Questionnaire version III [28, 29]. It consists of 3 items about feelings over the past four weeks (*i.e.*, “How often have you felt worn out?”, “How often have you been physically exhausted?”, “How often have you been emotionally exhausted?”). Responses were given on a 5-point scale ranging from “not at all” to “all the time” and scored from 0 to 100 in steps of 25 according to Berthelsen et al. [29]. A total score was calculated as the mean of the item responses if at least two of the items had been responded to. The internal consistency of the scale, in terms of Cronbach’s alpha in the sample, was 0.83 at baseline and 0.83 at follow-up.

### Independent variables

#### Telework mismatch

In the questionnaire, the item used to assess the actual extent of telework was “How often do you telework?”. The item used to assess the respondent’s preferred extent of telework was “If you had the choice, to what extent would you telework in the future?”. For each of the two items, responses were given in days per week (0–7). Telework mismatch was calculated as the difference between the actual and preferred extent of telework and respondents were categorized into three groups: those who teleworked exactly as much as they would like, those who teleworked less than they would like, and those who teleworked more than they would like.

#### Change in telework mismatch

The difference between actual and preferred extent of telework was calculated as explained above from questionnaire responses at baseline as well as follow-up. From the responses, three different groups of respondents were identified: those who experienced less mismatch at follow-up than at baseline, those who experienced more mismatch at follow-up, and those who had the same level of mismatch at baseline and follow-up. A change in mismatch between baseline and follow-up may be due to changes in either actual or preferred extent of telework or both.

### Covariates

Based on previous literature on telework, the following covariates were adjusted for in the analysis: age, sex, marital status, children, type of employment, commuting time, and extent of telework performed [9–11, 14]. Commuting time, *i.e.*, time spent traveling to work, was assessed in minutes. Due to its skewed distribution, it was dichotomized into 0–29 min and ≥ 30 min [30].

### Statistical analysis

All analyses were performed in IBM SPSS Statistics version 27 for Windows (IBM Corp., Armonk, NY, USA). Descriptive statistics of participants’ characteristics are presented as proportions, means and standard deviations (SD). For research question 1, the baseline questionnaire responses were analyzed using multivariate and univariate analyses of variance with well-being and burnout as dependent variables and telework mismatch as independent variable. For research question 2, multivariate and univariate repeated measures analyses of variance were performed with well-being and burnout as dependent variables, and time (*i.e.*, baseline and follow-up) and telework mismatch at baseline as independent variables.

To determine whether change in mismatch over time was associated with changes in ratings of well-being and burnout (*i.e.*, research question 3), multivariate and univariate repeated measures analyses of variance were performed with well-being and burnout as dependent variables. Since eight of nine combinations of the variables telework mismatch at baseline and change in mismatch over time exist (the non-existing combination being no mismatch at baseline and less mismatch at follow-up), a categorical variable was created that consisted of the 8 combinations between level of mismatch at baseline and change in mismatch over time. This variable was used as independent variable in the analyses of variance, in addition to time (*i.e.*, baseline and follow-up).

All analyses were performed with and without adjustment for age, sex, marital status, children, type of employment, commuting time and extent of telework performed. In the analyses of variance, *p* < 0.05 was considered significant and model assumptions were checked using Box’s M test, Levene’s test, and standard graphical procedures.

## Results

At baseline, 928 employees responded to the questionnaire, corresponding to a response rate of 39%. Among these respondents, 556 employees responded to the questionnaire at follow-up and 401 of them provided ratings of actual and preferred extent of telework on both occasions. Table 1 shows the respondents’ characteristics at baseline and follow-up. The age did not differ between respondents and non-respondents at baseline (mean 45 years in both groups) and the extent of employment was similar between the groups (97% and 98% full-time employees, respectively). However, the proportion of women and permanently employed was higher among respondents than among non-respondents (33% versus 23% women, and 90% versus 78% permanently employed). At follow-up, the mean age (assessed at baseline) of the 556 respondents was 46 years and for non-respondents 44 years. The extent of employment (assessed at baseline) did not differ between respondents and non-respondents (97% full-time employees), but the proportion of women and permanently employed (assessed at baseline) was higher among respondents than among non-respondents (36% versus 29% women, 94% versus 83% permanently employed).

**Table 1:** Characteristics of the respondents at baseline and follow-up

		Baseline (*n* = 928)			Follow-up (*n* = 556)		
		Proportion	Mean	SD	Proportion	Mean	SD
Age (years)^a^			45	11		46	11
Sex^a^	Man	67			64		
	Woman	33			36		
Marital status^a^	Living alone	20			19		
	Living with partner	80			81		
Children 0–12 years at home^a^		35			34		
Type of employment^a^	Permanent	90			94		
	Other	10			6		
Extent of employment^a^	Full time	97			97		
	Part time	3			3		
Commuting time^a^	0–29 min	75			77		
	≥ 30 min	25			23		
Prevalence of telework		77			86		
Extent of telework (days/week)			2.8	2.1		3.5	2.0
Preferred extent of telework (days/week)			2.3	1.6		2.6	1.6
Well-being (0–100)			57	20		59	19
Burnout (0–100)			33	21		33	21

*SD* standard deviation

^a^Assessed at baseline

### Telework mismatch at baseline

Most of the employees teleworked more than they would like (*n* = 336), followed by those who teleworked exactly as much as they would like (*n* = 246) and those who teleworked less than they would like (*n* = 160). Employees with no mismatch at baseline teleworked on average 2.2 days per week. The average mismatch among employees who teleworked less than they would like was 1.9 days (SD 1.2), *i.e.*, they would prefer to telework 1.9 days more than they did. Among employees who teleworked more than they would like, the mismatch was -2.0 days (SD 1.2), *i.e.*, they were teleworking 2.0 days more than they preferred. The multivariate analysis of variance showed a significant multivariate effect of the mismatch groups on well-being and burnout (*p* = 0.007, Table 2). The effect remained when the analysis was adjusted for age, sex, marital status, children at home, type of employment, commuting time and extent of telework performed (Table 2). Among the covariates, age, marital status and type of employment were significant.

**Table 2:** Telework mismatch versus well-being and burnout at baseline. Results from multivariate and univariate analysis of variance with and without adjustment for covariates (listwise *n* = 688)

	Multivariate analysis of variance				Univariate analyses of variance			
					Well-being		Burnout	
	Pillai’s trace	F	P	Partial η^2^	P	Partial η^2^	P	Partial η^2^
*(Unadjusted model)*								
Telework mismatch	**0.020**	**3.57**	**0.007**	**0.010**	**0.019**	**0.011**	0.623	0.001
*(Adjusted model)*								
Telework mismatch	**0.018**	**3.12**	**0.014**	**0.009**	**0.012**	**0.013**	0.816	0.001
Age	**0.020**	**7.04**	**0.001**	**0.020**	0.731	< 0.001	**0.002**	**0.014**
Sex	0.006	2.17	0.115	0.006	0.050	0.006	0.460	0.001
Marital status	**0.010**	**3.38**	**0.035**	**0.010**	**0.015**	**0.009**	0.366	0.001
Children	0.001	0.17	0.841	0.001	0.646	< 0.001	0.563	< 0.001
Employment	**0.013**	**4.55**	**0.011**	**0.013**	**0.006**	**0.011**	**0.006**	**0.011**
Commuting	0.006	1.17	0.115	0.006	0.397	0.001	**0.046**	**0.006**
Extent of telework	0.003	1.06	0.347	0.003	0.233	0.002	0.889	< 0.001

Significant differences are shown in bold. P values and effect size in terms of partial eta squared are presented for the multivariate as well as the univariate analyses of variance. Telework mismatch (3 categories: respondents who teleworked more than they would like, respondents who teleworked as much as they want, respondents who did not telework as much as they would like); Age (years); Sex (woman); Marital status (living alone); Children = children 0–12 years at home; Employment = type of employment (permanent position); Commuting = commuting time ≥ 30 min; Extent of telework = extent of telework performed (days per week)

**Fig. 1:**
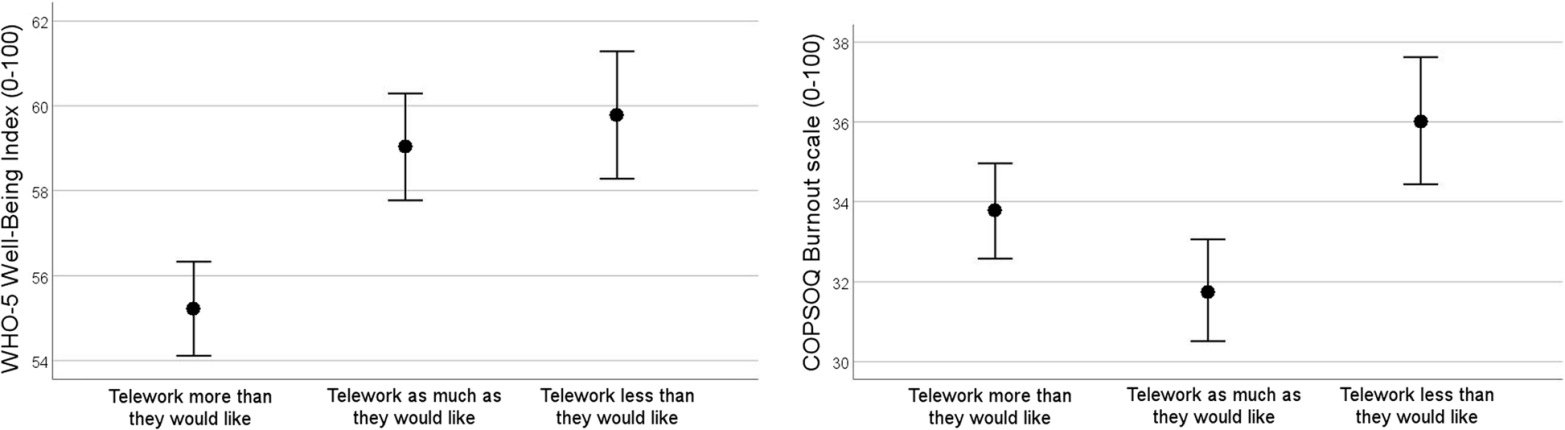
Descriptive mean values of well-being and burnout at baseline. Higher values represent better well-being and more burnout, respectively. Error bars represent ± 1 standard error of the mean

Separate analyses of variance for well-being and burnout showed significant differences between the telework mismatch groups in well-being (*p* = 0.019, Table 2) but not in burnout (*p* = 0.623, Table 2). Tukey’s HSD pairwise post-hoc tests showed that those who teleworked more than they would like reported less well-being than those who teleworked less than they would like (*p* = 0.047, Fig. 1). The pairwise difference between those who teleworked more than they would like and those who teleworked as much as they would like was not significant (*p* = 0.058, Fig. 1). Mean (SD) for well-being was 55.2 (19.8) for those who teleworked more than they would like, 59.0 (19.2) for those who teleworked exactly as much as they would like, and 59.8 (18.3) for those who teleworked less than they would like. For burnout, the corresponding values were 33.8 (21.1), 31.7 (20.7) and 36.0 (20.2).

When adjusting the analyses of variance for age, sex, marital status, children at home, type of employment, commuting time and extent of telework performed, the difference between the telework mismatch groups in well-being remained (*p* = 0.012, Table 2). Among the covariates, marital status and type of employment were significantly associated with well-being (*i.e.*, well-being was worse among those who lived alone and those who were permanently employed), and age, type of employment and commuting time were significantly associated with burnout (*i.e.*, less burnout with age, more burnout among those who were permanently employed and those who had < 30 min commuting time).

### Prediction of well-being and burnout

The multivariate repeated measures analysis of variance showed no significant effect of telework mismatch at baseline on changes in well-being and burnout over 10 months (*p* = 0.189, Table 3). Similar results were found in the univariate repeated measures analyses of variance for well-being and burnout, and did not change when the models were adjusted for the covariates (Table 3). However, actual extent of telework performed at baseline was associated with change in burnout (*i.e.*, more telework at baseline predicted decreased burnout after 10 months, *p* = 0.011).

**Table 3:** Telework mismatch versus well-being and burnout over time. Results from multivariate and univariate repeated measures analysis of variance with and without adjustment for covariates (listwise *n* = 414)

	Multivariate analysis of variance				Univariate analyses of variance			
					Well-being		Burnout	
	Pillai’s trace	F	P	Partial η^2^	P	Partial η^2^	P	Partial η^2^
*(Unadjusted model)*								
Time	**0.020**	**4.24**	**0.015**	**0.020**	**0.006**	**0.018**	0.568	0.001
Time^a^Telework mismatch	0.015	1.54	0.189	0.007	0.193	0.008	0.089	0.012
*(Adjusted model)*								
Time	0.002	0.49	0.611	0.002	0.677	< 0.001	0.548	0.001
Time^a^Telework mismatch	0.003	0.35	0.847	0.002	0.902	0.001	0.706	0.002
Time^a^Age	0.001	0.25	0.780	0.001	0.497	0.001	0.876	< 0.001
Time^a^Sex	0.002	0.30	0.738	0.002	0.489	0.001	0.521	0.001
Time^a^Marital status	0.002	0.48	0.619	0.002	0.392	0.002	0.985	< 0.001
Time^a^Children	0.003	0.68	0.505	0.003	0.527	0.001	0.244	0.003
Time^a^Employment	0.003	0.66	0.519	0.003	0.412	0.002	0.750	< 0.001
Time^a^Commuting	0.006	1.30	0.274	0.006	0.444	0.001	0.108	0.006
Time^a^Extent of telework	**0.016**	**3.27**	**0.039**	**0.016**	0.150	0.005	**0.011**	**0.016**

^a^indicates interaction between the variables. Significant differences are shown in bold. *P* values and effect size in terms of partial eta squared are presented for the multivariate as well as the univariate analyses of variance. Time (baseline, follow-up); Telework mismatch (3 categories: respondents who teleworked more than they would like, respondents who teleworked as much as they want, respondents who did not telework as much as they would like); Age (years); Sex (woman); Marital status (living alone); Children = children 0–12 years at home; Employment = type of employment (permanent position); Commuting = commuting time ≥ 30 min; Extent of telework = extent of telework performed (days per week)

### Change in telework mismatch

**Fig. 2:**
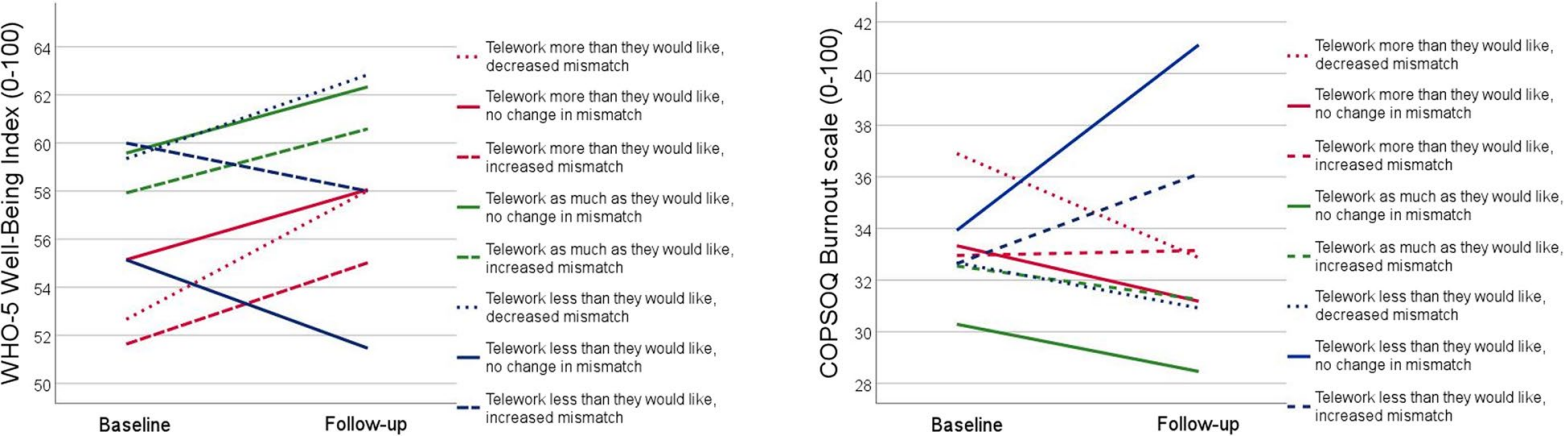
Descriptive mean values of well-being and burnout at baseline and follow-up for each of the eight combinations between mismatch at baseline (red, green and blue lines) and change in mismatch over time (dotted, dashed and solid lines). Higher values represent better well-being and more burnout, respectively

Table 4 shows descriptive statistics for each of the eight combinations between mismatch at baseline and change in mismatch over time. The multivariate and univariate repeated measures analyses of variance showed no significant effect of the combinations between level of mismatch at baseline and change in mismatch over time on well-being and burnout over 10 months (*p* = 0.741, Table 5 and Fig. 2). The results did not change when the models were adjusted for the covariates (Table 5).

**Table 4:** Descriptive statistics (assessed at baseline) for the combinations between mismatch at baseline (rows) and change in mismatch over time (columns). In total, 401 respondents provided ratings of actual and preferred extent of telework at baseline and follow-up

	Increased mismatch at follow-up		No change in mismatch at follow-up		Decreased mismatch at follow-up	
Telework more than they would like at baseline	*n* =	45	*n* =	63	*n* =	93
	Age =	47(11)	Age =	46(10)	Age =	46(11)
	60% men		57% men		56% men	
	7% living alone		13% living alone		22% living alone	
	36% children at home		43% children at home		39% children at home	
	100% permanently employed		92% permanently employed		94% permanently employed	
	84% < 30 min commuting time		70% < 30 min commuting time		78% < 30 min commuting time	
	Extent of telework =	3.9(1.3)	Extent of telework =	4.3(1.0)	Extent of telework =	4.5(1.1)
	Preferred extent of telework =	2.2(1.2)	Preferred extent of telework =	2.5(1.1)	Preferred extent of telework =	2.0(1.2)
Telework as much as they would like at baseline	*n* =	56	*n* =	78		
	Age =	45(11)	Age =	46(10)		
	71% men		77% men			
	20% living alone		23% living alone			
	38% children at home		33% children at home			
	98% permanently employed		92% permanently employed			
	82% < 30 min commuting time		62% < 30 min commuting time			
	Extent of telework =	2.3(1.7)	Extent of telework =	2.7(2.1)		
	Preferred extent of telework =	2.3(1.7)	Preferred extent of telework =	2.7(2.1)		
Telework less than they would like at baseline	*n* =	12	*n* =	15	*n* =	39
	Age =	46(9)	Age =	44(13)	Age =	42(11)
	50% men		67% men		67% men	
	0% living alone		33% living alone		18% living alone	
	42% children at home		33% children at home		31% children at home	
	100% permanently employed		87% permanently employed		95% permanently employed	
	83% < 30 min commuting time		80% < 30 min commuting time		77% < 30 min commuting time	
	Extent of telework =	1.1(1.2)	Extent of telework =	0.5(1.2)	Extent of telework =	1.2(1.4)
	Preferred extent of telework =	2.4(1.2)	Preferred extent of telework =	2.5(1.4)	Preferred extent of telework =	3.2(1.6)

Number, proportion and mean (standard deviation) at baseline. Age (years); Children at home = children 0–12 years at home; Extent of telework and Preferred extent of telework (days per week)

**Table 5:** Change in telework mismatch versus well-being and burnout over time. Results from multivariate and univariate repeated measures analysis of variance with and without adjustment for covariates (listwise *n* = 369)

	Multivariate analysis of variance				Univariate analyses of variance			
					Well-being		Burnout	
	Pillai’s trace	F	P	Partial η^2^	P	Partial η^2^	P	Partial η^2^
*(Unadjusted model)*								
Time	0.012	2.25	0.107	0.012	0.072	0.009	0.915	< 0.001
Time^a^Combination	0.028	0.73	0.741	0.014	0.487	0.017	0.347	0.021
*(Adjusted model)*								
Time	0.033	1.95	0.147	0.033	0.068	0.029	0.805	0.001
Time^a^Combination	0.095	0.81	0.653	0.048	0.419	0.059	0.928	0.021
Time^a^Age	0.036	2.08	0.130	0.036	0.056	0.032	0.726	0.001
Time^a^Sex	< 0.001	0.01	0.989	< 0.001	0.943	< 0.001	0.883	< 0.001
Time^a^Marital status	0.014	0.82	0.441	0.014	0.510	0.004	0.521	0.004
Time^a^Children	0.029	0.41	0.912	0.014	0.835	0.013	0.696	0.019
Time^a^Employment	0.016	0.90	0.411	0.016	0.349	0.008	0.705	0.001
Time^a^Commuting	0.034	2.01	0.139	0.034	0.134	0.020	0.670	0.002
Time^a^Extent of telework	< 0.001	0.02	0.984	< 0.001	0.911	< 0.001	0.946	< 0.001

^a^indicates interaction between the variables. Significant differences are shown in bold. *P* values and effect size in terms of partial eta squared are presented for the multivariate as well as the univariate analyses of variance. Time (baseline, follow-up); Combination (8 categories: combinations between level of mismatch at baseline and change in mismatch over time); Age (years); Sex (woman); Marital status (living alone); Children = children 0–12 years at home; Employment = type of employment (permanent position); Commuting = commuting time ≥ 30 min; Extent of telework = extent of telework performed (days per week)

### Model assumptions

In the multivariate analyses of variance, Box’s M test supported the assumption that the within-group covariance matrices were equal. In the univariate analyses of variance, equal variance across the categories of respondents could be assumed for both dependent variables (Levene’s test: *p* > 0.218). When inspected, the residuals did not show large deviations from normality (skewness: -0.54 – 0.74; kurtosis: -0.47 – 0.63).

## Discussion

The present study aimed to determine whether telework mismatch, *i.e.*, lack of fit between actual and preferred extent of telework, is associated with well-being and burnout. A cross-sectional association was found between telework mismatch and well-being, showing that employees who teleworked more than they would like reported worse well-being than those who teleworked less than they would like. No statistically significant association was found between telework mismatch and burnout. The ability of telework mismatch at baseline to predict changes in well-being or burnout over 10 months was small and non-significant. When change in telework mismatch over 10 months was analyzed, we found no associations with changes in well-being or burnout.

Our finding that employees who teleworked more than they would like reported worse well-being than those who teleworked less than they would like, while they had almost the same size of mismatch (1.9 days versus 2.0 days), suggests that the direction of the mismatch is of importance. This result is in agreement with Otsuka et al. [20], who found that the association between extent of telework and psychological distress differed depending on telework preference. Further, Oakman et al. [31] found that working from home more than preferred during the Covid-19 pandemic was associated with increased stress and a larger likelihood of reporting musculoskeletal pain among employees older than 45 years. Similar findings have been reported in studies of voluntary versus involuntary telework prior to the pandemic [32, 33]. When Kaluza and van Dick [34] compared data collected before and during the pandemic, they found that employees experienced fewer disadvantages with telework (*e.g.*, social isolation, poor work-life balance, distractions) the more telework they performed, but the finding only applied to individuals experiencing a high degree of voluntariness of the telework arrangement. Taken together, it appears that the autonomy associated with telework arrangements is crucial for the individual experience. Arguably, autonomy in terms of deciding when and where to telework would benefit employees regardless of their individual conditions for telework, such as having access to an office space at home.

Although telework mismatch was associated with well-being at baseline, we found no effect of telework mismatch at baseline or change in telework mismatch from baseline to follow-up on either well-being or burnout over 10 months. In a study based on data collected prior to the pandemic, De Wind et al. [21] also found a cross-sectional association between mismatch (*i.e.*, employees’ access to versus need for working from home) and work-home interference and fatigue. Neither the present study nor the De Wind et al. study [21] found associations between mismatch at baseline and changes in outcomes after one year, but the results may depend on the change in mismatch over time. However, when a 10-month change in telework mismatch was investigated in the present study, we found no indication that it was associated with changes in well-being or burnout. Due to the small number of respondents in some of the groups (*e.g.*, employees who teleworked less than they would like at baseline and experienced more mismatch at follow-up), this result should be interpreted with caution.

Of the two outcomes in this study, only well-being was associated with telework mismatch. Previous studies have shown associations between well-being and burnout [35, 36], but findings may differ depending for example on how burnout is assessed. The burnout scale in the Copenhagen Psychosocial Questionnaire version III, which was used in this study, focus exclusively on exhaustion (see items in the Methods section). According to Angelini et al. [37], exhaustion is a central part of burnout. In a recent review of burnout studies, however, Edú-Valsania et al. [38] found that emotional exhaustion is the more common among women while depersonalization occurs more among men. Considering that our sample consisted mostly of men, this may be reflected in the ratings of burnout, and may potentially explain the non-significant findings.

The results found in this study partly supports the person-environment fit theory. Employees who perceived lack of fit to telework practice reported worse well-being if they had more telework than they preferred. This may be a result of feeling trapped in a work form that does not suit you. In this study, a majority of the respondents teleworked to some extent (77% at baseline, see Table 1), and teleworking was unevenly distributed across the mismatch groups. Among those who preferred less telework, 96% teleworked at least during two days per week, whereas among those who preferred more telework, 56% did not telework at all. One may argue that those who cannot telework but would like to do so are also trapped in a work form that does not suit them. Still, effects on well-being were different between those who teleworked more than they would like and those who teleworked less than preferred. Since the effects were found in cross-sectional analyses, it is also possible that employees with poor wellbeing tended to experience a larger mismatch. To better understand the effect of imposed telework on well-being, ratings of well-being should be compared between those who have to telework despite preferring not to, those who are not allowed to telework despite preferring to, and those who decide for themselves how much they telework, but that is beyond the scope of this study.

While lack of fit in telework practice changed over time in our sample, we did not find any effect of it on changes in well-being or burnout. It is possible that the employees adapted to the situation during the pandemic, and therefore experienced the same level of well-being at baseline and follow-up (see Table 1 for averages across the sample). It is also possible that ratings of well-being were affected by an awareness that COVID-19 related recommendations would eventually come to an end, particularly at follow-up when vaccine coverage for COVID-19 was getting high. Larger studies are needed to determine the effects of lack of fit to telework practice on employee well-being over time.

Among the covariates adjusted for in the baseline analyses, type of employment was associated with both well-being and burnout. Employees who were permanently employed, *i.e.*, a majority of the sample, rated worse well-being and more burnout than employees with other types of employment, such as temporary employment. A similar finding was reported in a study on telework among academics [11], who found that academics with a permanent position reported more stress than academics with a temporary position. In the present study, older age was associated with less burnout, which may be related to, *e.g.*, higher levels of skill and decision authority at work [39]. Living with a partner was associated with better well-being. Similar findings have been reported in several previous studies [11, 40, 41]. In the prospective analyses, extent of telework was associated with reduced burnout over 10 months. Possibly, the finding is affected by the work tasks performed. Windeler et al. [42] found that teleworking from home was longitudinally associated with reduced work exhaustion due to reduced interpersonal interaction (*i.e.*, interacting and engaging with others at work), but with increased work exhaustion due to external interaction (*i.e.*, interacting with stakeholders external to one’s business unit).

The extent of telework performed by the employees varied within and between the mismatch groups (see Table 4). The group that reported no mismatch at baseline teleworked approximately 2.2 days per week, but the variation within the group was high (coefficient of variation: 88%). In the other groups, the average preferred extent of telework ranged between 2.0 and 3.2 days per week. Over the 10-month follow-up period, the change in respondents’ preferred extent of telework was small relative to the change in extent of telework performed (see Table 1). This may imply that the main reason for the change in mismatch was that telework was practiced to a larger extent at follow-up. While group statistics may guide the implementation of telework in companies or organizations, the findings from this study imply that careful implementation is needed to avoid negative consequences for some employees. In particular, reasons for differences between individuals in their preferences regarding telework need to be elucidated.

### Limitations

The study is based on data collected in one company, which may limit generalizability of the findings. At the same time, the findings may be considered representative of working life after the COVID-19 pandemic, since no strict COVID-19 regulations such as lockdowns were imposed that would additionally affect the well-being among employees. The response rate at baseline was low, despite several measures taken to improve it. Further, ratings of well-being and burnout differed between employees with different types of employment, and the sample contained a larger proportion of permanently employed than the population. Thus, in that respect, the sample is not fully representative of the population. In this study, it was not possible to determine whether ratings of preferred extent of telework were affected by the employees’ perception of how and where the work can be performed, *e.g.*, if they depended on the employees’ work tasks and living situation. Further, change in actual and preferred extent of telework is related to baseline estimates. If the extent of telework is very high or very low, there is little room for change in ratings upwards and downwards, respectively.

Since telework will likely remain a regular feature of work for many employees, studies are needed of the long-term effects of practicing it. In large samples it may be possible to determine how long-term changes in match between preferred and actual extent of telework is associated with employee health and well-being, including how the associations are modified by the nature of the job and the work environment. It is also important to understand how telework is distributed among the employees and how different principles of doing that affects organizational performance.

## Conclusion

The present study showed that mismatch between the actual extent of telework performed and the preferred extent of telework was associated with poor well-being, but only if telework was practiced to a larger extent than preferred. We found no effect of telework mismatch at baseline or change in mismatch over a 10-month period on changes in ratings of well-being and burnout. Our results suggest that telework should be thoughtfully practiced in companies or organizations to avoid negative consequences for employees who already telework more than they prefer. Studies are needed to determine how long-term changes in match between preferred and actual extent of telework is associated with employee health and well-being, including how the associations are modified by the nature of the job and the work environment.

## Data Availability

The datasets used and/or analyzed during the current study are available from the corresponding author on reasonable request.
